# Social optimism biases are associated with cortical thickness

**DOI:** 10.1093/scan/nsaa095

**Published:** 2020-07-18

**Authors:** Dominik Andreas Moser, Mihai Dricu, Roland Wiest, Laurent Schüpbach, Tatjana Aue

**Affiliations:** University of Bern, Institute of Psychology, Bern 3012, Switzerland; University of Bern, Institute of Psychology, Bern 3012, Switzerland; Institute of Diagnostic and Interventional Neuroradiology, Bern 3010, Switzerland; University of Bern, Institute of Psychology, Bern 3012, Switzerland; University of Bern, Institute of Psychology, Bern 3012, Switzerland

**Keywords:** social optimism bias, cortical thickness, gray matter, MRI, stereotyping

## Abstract

Optimism biases denote the tendency to see future desirable events as being more likely to happen to oneself than undesirable events. Such biases are important for mental health and may extend to other individuals or social groups (social optimism biases). However, little is known about whether social optimism biases relate to brain structure. Using sparse canonical correlation analysis, we associated cortical thickness (assessed by magnetic resonance imaging) with measures of social and personal optimism bias, trait optimism and related concepts. We identified a defensive self-enhancement dimension that associated significantly and reliably with the cortical thickness of the insula and inferior frontal cortex. This self-enhancement dimension included unfavorable biases toward unpopular out-groups and indicators of personal optimism and pessimism. A shared biological substrate underlying future expectancies that subserves the promotion of the self and the denigration of unpopular out-groups may render society-wide efforts to counteract stereotyping particularly difficult: such efforts may hinder the establishment of adaptive personal optimism biases.

## Introduction

Optimism bias can be defined as the tendency to expect positive outcomes to be more likely than negative outcomes (Krizan and Windschitl, 2007; Lench and Ditto, 2008; Lench and Bench, 2012; Dricu *et al*., 2018). Most people display exaggerated optimism about their own future. This self-centered overoptimism has been termed optimism bias in the literature (Windschitl and Stuart, 2015) and has been suggested as a prerequisite for mental health (Trimmer, 2016). Interestingly, such unrealistic optimism extends toward in-groups and individuals that one evaluates positively or identifies with (we refer to this extension of optimism toward others as social optimism bias). Accordingly, sports fans are overly optimistic about their favorite team winning the game (Price, 2000; Aue *et al*., 2012), and voters overestimate the chances of their preferred political candidate winning elections (Babad, 1997).

While there is a general tendency toward unrealistic optimism in the population, there is also interindividual variability in optimism bias and its related concepts. For instance, an individual’s readiness to display (social) optimism bias is likely influenced by dispositional optimism, a relatively stable disposition of having an optimistic yet not necessarily unrealistic life orientation (Radcliffe and Klein, 2002; Shepperd *et al*., 2015). Correspondingly, one may expect some overlap in neural correlates between personal and social optimism biases. Personal optimism biases and trait optimism have been linked to the ventromedial prefrontal cortex (vmPFC) and the anterior cingulate cortex (ACC), brain regions involved in self-referential processing and stimulus valuation (Blair *et al*., 2013; Kuzmanovic *et al*., 2016). Interestingly, activation of the aforementioned regions is also enhanced while an individual deliberates over in-group members (Volz *et al*., 2009; Cikara *et al*., 2017). Beyond these regions, the orbitofrontal cortex (OFC), posterior cingulate cortex (PCC) and insula have been associated with personal optimism bias (Blair *et al*., 2013; Kuzmanovic *et al*., 2016). Finally, the sole functional magnetic resonance imaging (fMRI) study that focused on social optimism bias identified similar regions: social optimism bias in American football fans was characterized by enhanced positive connectivity between the primary visual cortex on the one hand and the insula, PCC and striatum on the other (Aue *et al*., 2012).

Whether and how unrealistic optimism manifests toward out-group members depends on the stereotypical characteristics assigned to them. When specifying the likelihood of different events happening to both in-group characters and different out-group characters, participants revealed equally strong optimism biases toward the in-group as they did toward an out-group that was perceived as warm but low competent [i.e. elderly persons; (Dricu *et al*., 2018)]. By contrast, an out-group that was perceived as cold and competent (i.e. businesspersons) was associated with substantially reduced social optimism bias. Finally, the effect was flipped into pessimism bias toward an out-group that was perceived as most distant from our participants and was also cold and low competent (i.e. alcoholic persons). A similar study (Dricu *et al*., 2020) that used the same student sample as in the present study but that focused on brain *activation* has linked the vmPFC/ACC and the PCC to differences in social optimism biases between the in-group and three different types of out-groups (elderly persons, businesspersons and alcoholic persons, representing progressing social distance; cf. Dricu *et al*., 2018). Biases toward out-groups also correlated with activation in the insula and the inferior frontal gyrus (IFG).

Anatomical characteristics of brain structures involved in optimism bias and associated concepts have just started to be identified. Trait optimism, for instance, has been linked to increased volume of the left OFC, thalamus (Dolcos *et al*., 2016) and parahippocampus (Yang *et al*., 2013). Associations between brain structure and expressions of social optimism bias have not yet been investigated, and little is known about the relationship between personal optimism bias and brain structure. Studies on optimistic belief update bias may come closest. This type of bias refers to the phenomenon whereby people more readily update likelihood estimates in response to favorable rather than unfavorable feedback regarding their personal future (thus it is related to personal optimism bias). Chowdhury and colleagues (Chowdhury *et al*., 2014) found that ACC volume in healthy older adults, but not among younger adults, correlated with the magnitude of optimistic update bias. Furthermore, white matter connectivity from the IFG and the insula to the subcortex was associated with the size of the belief update bias (Moutsiana *et al*., 2015).

In summary, in the literature on personal optimistic bias, the insula, the ACC and the IFG stand out as those regions most reliably associated with personal optimism bias (Harris and Fiske, 2007; Sharot *et al*., 2007; Gozzi *et al*., 2009; Singer *et al*., 2009; Lamm *et al*., 2011; Sharot *et al*., 2012; Blair *et al*., 2013; Marcoux *et al*., 2013; Blair *et al*., 2017; Kuzmanovic *et al*., 2018; Dricu *et al*., 2020). They are also implicated in social group perception (Krill and Platek, 2009; Hein *et al*., 2010; Harris and Fiske, 2011; Scheepers *et al*., 2013) and are therefore most likely to be involved in social optimism bias.

Given the impact of likelihood estimations in social contexts on some of our most important decisions (e.g. with whom to start a company), the lack of research on neural substrates of social forms of optimism bias represents an important shortcoming. While plastic, brain structures do not change as quickly as brain activations. If social optimism biases relied on particular brain structures, likely those biases would be hard to overcome, which should affect society at large. The present study, therefore, had three aims: (1) identify potential gray matter correlates of different social optimism biases; (2) examine their overlap with personal optimism bias; and (3) examine the overlap between biases and related concepts (e.g. trait optimism).

**Fig. 1:**
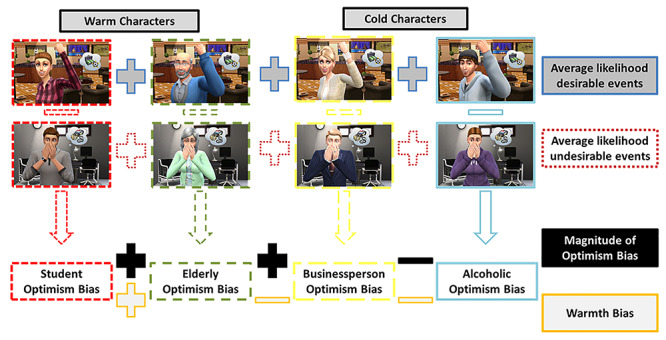
Image of all eight included social optimism bias measures. One optimism bias was computed for each character as the difference between the average likelihood of desirable events and the average likelihood of undesirable events. The warmth bias indicates the difference in optimism biases between warm (student, elderly) and cold (businessperson, alcoholic) characters. The magnitude of the optimism biases adds the optimism biases of the student, elderly and businessperson characters and then subtracts the optimism bias of the alcoholic character, since that one—on average—is negative (i.e. is a pessimism bias). In addition, the average likelihoods were taken for all desirable and all undesirable events.

The task to measure social optimism biases asked student participants inside an MRI scanner to rate the likelihood that four gender-congruent fictional characters would experience a series of identical desirable and undesirable events. One character represented the in-group (student), and the other three characters (elderly, businessperson, alcoholic) represented out-groups of progressive social distance (cf. Dricu *et al*., 2018). We computed an optimism bias score for each of the four characters. Two characters were previously rated (Cuddy *et al*., 2007; Dricu *et al*., 2018) as being warm (e.g. relatable, friendly, honest) and two as being cold (e.g. unapproachable, unpredictable), allowing for the additional calculation of a warmth bias (i.e. social optimism bias for warm versus cold individuals). Another measure consisted of the magnitude of optimism biases across characters to determine the extent of overall biasing of social predictions. Figure 1 depicts all measures of social optimism bias included in this study. Together, the combination of these measures allowed us to estimate and quantify whether and how strongly different facets of social optimism biases are associated with cortical thickness. Because social optimism bias toward in-group members relies on identification processes, we hypothesized that the regions identified in fMRI research on personal optimism bias and social optimism biases (i.e. vmPFC/ACC, PCC/precuneus, insula) are also structurally different in participants with strong versus small social optimism biases. In addition, we expected that regions implicated in the perception of social groups [insula, ACC, IFG, dorsomedial PFC (dmPFC), temporoparietal junction] might be involved.

Because the task assesses social optimism biases, we wanted to complement our data set with measures of personal optimism bias, trait optimism and other measures potentially associated with social optimism bias (see Methods section for details). Measuring these related concepts allowed us to quantify their expression in brain structures as related to that of social optimism biases. Specifically, we were interested in finding out whether personal and social forms of optimism bias relied on the same brain structures, wherefore we added a measure of personal optimism (and pessimism) bias and a measure of trait optimism. In addition, we wanted to account for concepts that may be related but not identical to optimism. In that regard, we also measured the sensitivity of the behavioral inhibition system (BIS) and the behavioral activation system (BAS), which describe the regulation of behavior in response to punishment and reward, respectively (Carver and White, 1994; Strobel *et al*., 2001). Both systems might be closely associated with social forms of optimism bias (even though correlations with personal forms of optimism have been reported to be negative and moderate for inhibition [De Pascalis *et al*., 2013)]. This is because the predicted social outcomes may be evaluated with respect to the self and thereby perceived as either rewarding or threatening (Cuddy *et al*., 2007). Other concepts of relevance were emotion regulation strategies such as suppression and reappraisal (measured by the Emotion Regulation Questionnaire; ERQ), both of which appear to interact with dispositional optimism for the interpretation of ambiguous information (Gordon *et al*., 2016). Such an association might extend to the social context. Finally, we also included general affect [measured by the Positive and Negative Affect Schedule (PANAS); (Watson *et al*., 1988; Krohne *et al*., 1996)], self-esteem [Rosenberg Self-Esteem Scale (RSES); (Rosenberg, 1965; Von Collani and Herzberg, 2003)] and the Big-5 personality dimensions (Rammstedt and John, 2007), all of which might potentially affect likelihood estimations regarding success in others or oneself.

To achieve all of our aims, in terms of statistics we used sparse canonical correlation analysis (sCCA; see also Methods and Supplementary Information). This method uses an algorithm to assess the association between the different optimism measures (i.e. the non-imaging data) on the one hand and the different cortical thickness measures on the other. For this, the algorithm groups variables from either side into dimensions (modes). It also assigns weights to the variables constituting each mode in order to determine their respective contributions to the overall association between the non-imaging data and gray matter thickness.

## Methods

### Participants

Forty-nine healthy participants (20 male, age: *M* = 23.1 years; *SD* = 4.0 years; range = 19–36) were recruited at the University of Bern via e-mails, flyers and the local participant pool at the university. Participants were recruited in the context of a project on optimism biases, the fMRI-related parts of which were analyzed and described in a separate paper (Dricu *et al*., 2020). Here, we focus on brain *structure* instead of brain *activation*. Self-reported neurological disorders, mental disorders, MRI contraindications, use of psychoactive substances and left-handedness served as exclusion criteria. Participants had normal or corrected-to-normal vision and were reimbursed with course credits or 25 Swiss francs per hour. One participant was excluded because of the clearly reduced quality of segmentation as indicated by Computational Anatomy Toolbox (CAT 12) algorithms. One participant was excluded for failure to complete the questionnaires. The final analyses were performed on 47 participants.

### Method

#### Questionnaires

Using an online portal, participants filled out a number of questionnaire measures (see Supplementary Table S1A) prior to scanning. These measures included a sociodemographic questionnaire, the German version of the Comparative Optimism Scale [COS; measuring self-related future expectancies as compared with those for others; (Weinstein, 1980)], the Revised Life Orientation Test [LOT-R, measuring trait optimism; (Scheier *et al*., 1994; Glaesmer *et al*., 2008)], the ERQ [assessing different emotion regulation strategies, e.g. reappraisal and suppression; (Abler and Kessler, 2009)], the PANAS [assessing current affective state; (Watson *et al*., 1988; Krohne *et al*., 1996)], the 10-item Big Five Inventory (Rammstedt and John, 2007), the Behavioral Inhibition System and Behavioral Activation System Scales (BIS/BAS; measuring behavioral inhibition and behavioral approach; [Carver and White, 1994; Strobel *et al*., 2001)] and RSES [assessing personal self-esteem; (Rosenberg, 1965; Von Collani and Herzberg.Y, 2003)].

#### Social optimism bias task

The social optimism bias task required participants to give likelihood estimates for four fictional characters experiencing 16 desirable and 16 undesirable events (see Supplementary Figure S1 and Supplementary Table S2). Because of previous suggestions that perceived frequency and controllability of an event may be linked to altered optimism biases (Horswill and McKenna, 1999; Chambers *et al*., 2003; Klar and Ayal, 2004), we used validated events from a previous study (Dricu *et al*., 2018). Using a different sample and no brain imaging but the same stimuli and task as the present study, that study assessed events through ratings of perceived controllability (high and low) and perceived frequency (frequent and rare) with respect to the general population and ensured that they were matched across conditions (i.e. desirable and undesirable). Overall, 128 situations were created to reflect each character in each of the 32 events. Each trial started with a random-duration fixation cross (1.5–3 s), followed by a screen containing the target scenario. Scenarios consisted of a still animation in the middle of the screen, as well as a short description of the target event and a visual analog scale ranging from 0 to 100% at the bottom of the screen (see Figure 1 and Supplementary Figure 1). The trials were displayed in a pseudorandomized order (Latin square), divided into four acquisition blocks of 7 min.

The four animated characters represented social groups that would map onto each quadrant of the warmth–competence two-dimensional space as specified in the Stereotype Content Model (Cuddy *et al*., 2011; Fiske, 2015). A previous study that used the same characters had shown that students and elderly persons were indeed perceived as being warmer than businesspersons and alcoholic persons. Furthermore, students and businesspeople were perceived as being more competent than elderly persons and alcoholic persons (Dricu *et al*., 2018). Because we sampled student participants, the student character served as an implicit in-group character [i.e. perceived as being high in warmth and high in competence (Harris *et al*., 2000; Reynolds *et al*., 2000; Hogg and Reid, 2006)]. Three other—previously validated—characters represented out-groups: an elderly person (high warmth/low competence), a businessperson (high competence/low warmth) and an alcoholic person (low warmth/low competence). Thirty-two backgrounds pertinent to the target events were created onto which the four characters were carefully cropped by aligning size and position. All characters, backgrounds and scenarios were created by using *The Sims 4* (Electronic Arts, CA, USA). Stimuli were matched in brightness and contrast. The social optimism bias task was programmed with E-Prime 2.0 Professional (version 2.0.10.353; Psychology Software Tools, Pittsburgh, PA, USA). Female participants saw female animated characters and male participants viewed male animated characters.

#### Experimental procedure and MRI scan

Data collection took place at the Insel University Hospital of Bern, Switzerland. Participants first received a short booklet regarding the alleged nature of the study. The communicated purpose of the study was the investigation of the capacity to adequately think about the future. All procedures were approved by the ethics commission of the canton of Bern, Switzerland. After signing the consent form according to the guidelines of the Declaration of Helsinki, participants were placed inside the scanner.

MRI scans were conducted on a Siemens Magnetom Prisma scanner with a 64-channel head coil. The structural scan consisted of a T1-weighted protocol that acquired 160 interleaved slices, with a 1-mm isotropic spatial resolution, echo time of 2.98 ms, TR of 2.3 s, TI of 0.9 s, flip angle of 9 degrees and field of view of 256 × 256 × 160 mm.

### Analysis

#### Optimism bias task

We first engaged in data cleaning. The optimism bias task data of three participants who consistently gave extremely high or low (99 or 1%) or medium (50%) values were not considered. Medium values were excluded because 50% was the default value, and non-answers would have been registered as 50%. Thus, the default 50% value could reflect either genuine assessments of likelihood estimates or nonresponding. The present exclusion procedure accords with the methods of a previous study that identified participants whose proportion of such answers was more than three standard deviations above the average of the sample (Dricu *et al*., 2018). Approximately 1% of the trials from the remaining participants comprised no answer and were therefore excluded as well.

Next, we computed eight task measures for each participant (see Figure 1, Supplementary Table S1A). We averaged the likelihood estimates for desirable events across all characters (1) and did the same for undesirable events (2). We then estimated the optimism bias for each character (3–6) by (a) *z*-standardizing all likelihood values (across characters) within each participant and (b) subsequently computing the difference in likelihood estimates between desirable and undesirable events. In addition, we computed a warmth bias (7) by adding the optimism biases for the student and elderly characters and subtracting those of the businessperson and alcoholic characters. Finally, we computed the overall bias magnitude across characters (8) by adding the optimism biases for the student, elderly and businessperson characters, plus the inverted bias for the alcoholic character (as it was opposite in direction to the other biases in the vast majority of participants).

#### fMRI preprocessing and data extraction

MRI data were preprocessed and analyzed by using Statistical Parametric Mapping 12 software (Wellcome Department of Cognitive Neurology, London, UK; http://www.fil.ion.ucl.ac.uk/spm) implemented in MATLAB R2017b (Mathworks Inc., Sherborn, MA, USA). We determined cortical thickness with the CAT 12 toolbox, following the workflow specified in Dahnke et al. (Dahnke *et al*., 2013). We then extracted the mean cortical thickness for 62 regions (see Supplementary Table S1B) defined in the Desikan–Killiany–Tourville 40 atlas (Desikan *et al*., 2006; Klein and Tourville, 2012). We chose the measure of cortical thickness by using the DKT atlas in this parcellation because we deemed it a good compromise in terms of statistical power and regional granularity (spatial resolution) across the entire cortex. Analyses were performed on UBELIX (http://www.id.unibe.ch/hpc), the high-performance computing cluster at the University of Bern.

#### (Sparse) canonical correlation analysis

In order to associate the cortical thickness and the non-imaging data sets, we used a CCA that permits correlations between the variables within the two data sets. The purpose of CCA is to inform whether one data set associates with another. CCA specifies linear combinations (pairs of canonical variates) of variables in Data set 1 (non-imaging data) and variables in Data set 2 (cortical thickness data) that best express the maximal correlation (i.e. canonical correlation) between the two data sets. The correlations between the canonical variates are the canonical correlations. CCA can be considered a generalization of multiple linear regression with the advantage that it allows the combination of two data sets rather than a data set with one outcome variable. Our CCA approach used an L1 penalty function that does not assume that the variables within the two data sets are uncorrelated (Witten *et al*., 2009).

We applied sCCA (meaning that the weights of some variables can be zero) because this permitted the inclusion of more variables than participants but also because sCCA allows for stronger inferences regarding the contribution of individual variables [for further explanation of this and similar approaches, see (Moser *et al*., 2017; Moser *et al*., 2018)]. More in-depth description of the sCCA methods used can be found in the Supplementary Information.

We calculated the first 10 sCCA modes (components) to determine the variance explained. In short, each mode is the algorithm’s ideal representation of both data sets when the goal is to correlate Data set 1 linearly with Data set 2. Every mode is therefore represented by two variates: one that represents Data set 1 and one that represents Data set 2. Each mode does so without taking into account the variance explained by all previous modes. Therefore, the explained variance tends to get smaller with each subsequent mode. In (over-)simplified terms, a mode is essentially the equivalent of a factor in a factor analysis or a component in an independent component analysis, except that it takes two data sets simultaneously into account in order to create the modes.

Following the calculation of those 10 modes, we then restricted permutations and reliability analysis to the first seven modes, as they cumulatively explained more than 99% of the variance, meaning that any potentially explained variance by the remaining modes would have been small. We determined significance by using 10 000 permutations of the data set. For all modes, the threshold for statistical significance was set at *P* < 0.05. Next, for each mode, we extracted the weights of the variables contributing to both variates and reported variables with weights of more than 0.2 in the main text (and the others only in the Supplementary Data File, which is available at https://github.com/domamo/Supplementaries-Social-optimism-biases-are-associated-with-cortical-thickness). For those non-imaging measures whose contribution was in the top five significant modes, we performed post hoc correlations between the individual measures with cortical thickness across all 62 regions (corrected for height, weight, body mass index, age and sex), and we present regions that showed an uncorrected *P* < 0.001.

#### Reliability analyses

To ascertain that the sample size was adequate and that results from the sCCA were indeed reliable, we conducted three additional analyses, which are detailed more precisely in the Supplementary Information (Reliability Analysis section). In short, the reliability analysis included the following.

## Leave-one-out analyses for each participant

2a. Training-test set analyses. We randomly split the sample in half 10 000 times (resampling), performed sCCA on each of these training sets and then applied the identified weights from each training set to the other half of the sample.

2b. Mean and standard deviation of the redundancy–reliability score (RR-score) for each mode. The RR-score is a measure of the stability of the variable-to-variate correlations and essentially measures whether test sets have similar associations between variables and variates (Moser, 2018; Moser *et al*., 2018).

## We tested whether the sample size was adequate or would give rise to gross overfitting

*Post hoc analyses*: for the interested reader, we also provide uncorrected Pearson correlations between all non-imaging variables, including correlations of these variables with the sCCA variates of the first seven modes. The results can be found as part of the Supplementary Data File.

## Results

**Fig. 2:**
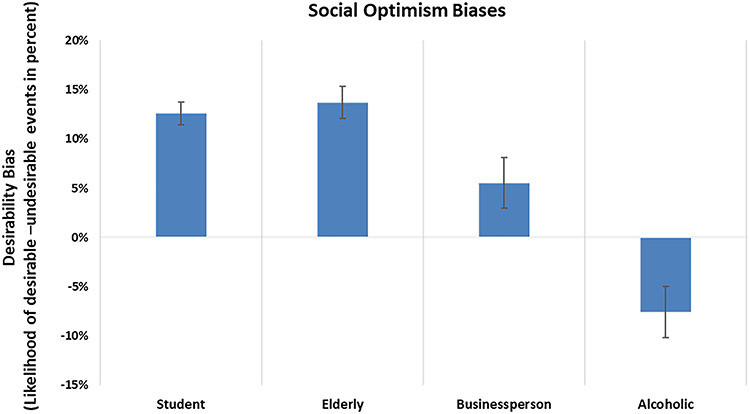
Optimism bias scores for the four social groups, calculated as the difference between the likelihood estimates for desirable events and undesirable events. The *y*-axis represents this desirability bias, as the percentage difference between likelihood estimates for desirable scenarios as opposed to undesirable scenarios. Error bars represent the standard error.

In line with a previous study (Dricu *et al*., 2018), the optimism biases were similarly positive for the student and the elderly characters, less positive for the businessperson and negative for the alcoholic character (see Figure 2).

### Sparse canonical correlation analysis

In terms of statistical inference for sCCA, the correlation between the non-imaging data and the cortical thickness variates reached significance for three modes: the first (*r* = 0.73, *P* = 0.0275), the second (*r* = 0.76, *P* = 0.0246) and the sixth (*r* = 0.78, *P* = 0.0185) modes. The remaining modes were not significant and are therefore not presented in the main manuscript (see Supplementary Data File).

**Fig. 3:**
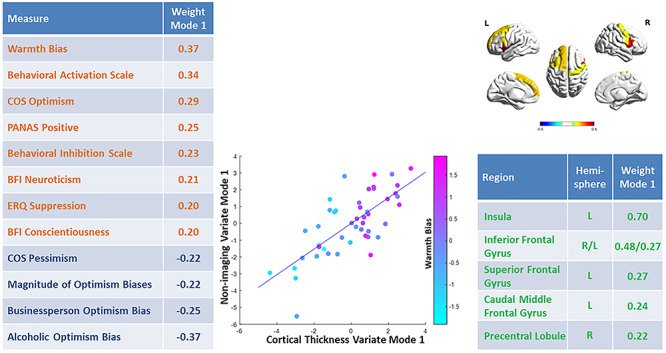
Depiction of results of the sparse canonical correlation analysis Mode 1. Left: list of all non-imaging variables with weights above 0.2 in descending order. Middle: scatterplot of both variates (*x*-axis, cortical thickness, *y*-axis, non-imaging data) colored by the *z*-standardized warmth bias. Top right: depiction of the weights of the brain regions with contributions toward the imaging variate above 0.2. Bottom right: list of these weights in descending order. Abbreviations: BFI = Big Five Inventory, COS = Comparative Optimism Scale, ERQ = Emotion Regulation Questionnaire, PANAS = Positive and Negative Affect Schedule, PFC = prefrontal cortex.

Numerous non-imaging variables contributed to Mode 1. Among variables emerging from the task, the warmth bias contributed positively toward the non-imaging variate (weight = 0.37), while the optimism biases for the alcoholic (−0.37) and the businessperson characters (−0.25) and the magnitude of the optimism bias (−0.22) contributed negatively to the non-imaging variate. Among questionnaire scales, the BAS (0.34) and the BIS (0.23), the COS Optimism (0.29), the PANAS Positive (0.25), the BFI Neuroticism (0.21) and Conscientiousness (0.20) and the ERQ Suppression (0.20) contributed positively to the non-imaging variate, while only the COS Pessimism subscale (−0.22) contributed negatively to the non-imaging variate (see Figure 3, Table 1, Supplementary Figure S4 and the Supplementary Data File).

**Table 1:** Contribution weights of all non-imaging measures toward the non-imaging variates of Modes 1, 2 and 6

	Weight mode 1	Weight mode 2	Weight mode 6
Task: warmth bias	0.37	0.18	0.22
BAS total	0.35	0.05	−0.12
COS optimism	0.29	−0.13	−0.13
PANAS positive	0.25	−0.11	0.01
BIS total	0.23	0.18	−0.40
BFI neuroticism	0.21	0.23	−0.04
ERQ suppression	0.20	−0.13	0.14
BFI conscientiousness	0.20	0.04	−0.25
ERQ reappraisal	0.19	0.24	0.16
Task: undesirable events likelihood average	0.18	0.21	−0.08
LOT optimism	0.09	−0.02	0.05
BFI Extraversion	0.05	−0.01	−0.07
Task: student optimism bias	0.01	0.13	0.37
Task: elderly optimism bias	0.00	0.16	0.22
RSES total	−0.03	0.20	0.27
PANAS Negative	−0.04	0.25	0.00
LOT pessimism	−0.04	−0.13	−0.35
BFI openness	−0.06	−0.16	0.01
BFI agreeableness	−0.11	0.01	−0.09
Task: desirable events likelihood average	−0.13	0.29	−0.01
COS pessimism	−0.22	0.39	−0.45
Task: business optimism bias	−0.23	−0.36	0.06
Task: overall magnitude of optimism bias	−0.25	0.41	0.15
Task: alcoholic optimism bias	0.37	−0.08	−0.08

*Note.* Abbreviations: BAS = Behavioral Activation System Scale, BFI = Big Five Inventory, BIS = Behavioral Inhibition System Scale, COS = Comparative Optimism Scale, ERQ = Emotion Regulation Questionnaire, LOT = Life Orientation Test, PANAS = Positive and Negative Affect Schedule, RSES = Rosenberg Self-Esteem Scale

Cortical thickness measures that contributed to the Mode 1 variate included the left insula (weight = 0.70), the right (0.48) and the left (0.27) IFG (pars opercularis), the left superior frontal gyrus (0.27) and the caudal middle frontal gyrus (0.24) and the right precentral gyrus (0.22; Figure 3).

Task variables that contributed positively to the Mode 2 non-imaging variate included the overall magnitude of the social optimism bias (weight = 0.41) and the average likelihood given to desirable (0.29) and undesirable (0.21) events. The businessperson optimism bias contributed negatively (−0.36) to the Mode 2 variate. Among questionnaire scales, the COS Pessimism (0.39), PANAS Negative (0.25), ERQ Reappraisal (0.24), BFI Neuroticism (0.23) and RSES (0.20) contributed positively to the Mode 2 non-imaging variate (Table 1, Supplementary Figures S1 and S3 and the Supplementary Data Set).

Cortical thickness measures that contributed positively to the Mode 2 non-imaging variate included the right rostral ACC (weight = 0.56), the PCC on both the left (isthmus, 0.53; above corpus callosum, 0.29) and the right (isthmus: 0.40) sides and the caudal ACC (0.31); the right paracentral gyrus (−0.24) contributed negatively (Supplementary Figure S2).

Task variables that contributed positively to the Mode 6 non-imaging variate included student (weight = 0.37) and elderly optimism biases (0.22). Among questionnaires, the RSES (0.27) contributed positively, while the COS Pessimism (−0.45), BIS (−0.40), LOT Pessimism (−0.35) and BFI Conscientiousness (−0.25) contributed negatively to the Mode 6 non-imaging variate (Table 1, Supplementary Figures S2 and S3 and Supplementary Data File).

All cortical thickness measures that contributed toward the Mode 6 non-imaging variate were located in the left hemisphere. The IFG (pars triangularis, weight = 0.66), the entorhinal gyrus (0.59) and the transverse temporal gyrus (0.24) contributed positively, while the only region that contributed negatively was the caudal middle frontal gyrus (−0.27).

### Reliability analyses

More in-depth description of the reliability analyses results can be found in the supplementary online materials. In short, we found that the leave-one-out approach indicated no high likelihood of outlier-dominated results. Test sets and RR-scores indicated high reliability for Mode 1 and reduced and low reliability for Modes 2 and 6, respectively. Sample size testing revealed only marginal overfitting, which indicates that the present sample size is sufficient to give reliable results with the present analysis (see Supplementary Figure S5).

## Discussion

In accordance with our aims, sCCA analysis indicated that (1) there were indeed gray matter thickness correlates of social optimism biases, (2) that these gray matter correlates of social optimism bias may partially overlap with similar correlates of personal optimism bias and (3) that there may be an interplay between the cortical gray matter correlate of optimism biases and related concepts. Specifically, we identified three sCCA modes that significantly related cortical thickness to measures of social and personal optimism bias and related concepts. On a general level, optimistic measures mostly contributed positively to Mode 1, while pessimism contributed negatively. Given that optimism (bias) has been linked to task persistence (Tenney *et al*., 2015) and lack of it is associated with pathologies centered around motivational problems (Garrett *et al*., 2014), Mode 1 cortical thickness could hence reflect preconditions for personal functionality and initiative. Consistent with this interpretation, both personal optimism bias (COS Optimism) and behavioral activation system sensitivity were characterized by positive weights on the Mode 1 cortical thickness variate.

Interestingly, regarding social optimism biases, the more negative the biases for the two ‘cold’ characters (businessperson and alcoholic), the greater the cortical thickness of these participants in brain regions identified with Mode 1. In that context, it is unsurprising that the bias difference for warm versus cold out-groups (i.e. the warmth bias) was also positively associated with cortical thickness. Thus, the businessperson and alcoholic character optimism biases were more similar to comparative pessimism than optimism (i.e. personal pessimism rather than optimism bias as measured with the COS) on this mode. This observation can be easily reconciled with findings suggesting that processes that act to decrease the impact of potentially self-threatening materials are involved in this mode, as suggested by the positive contribution of the BIS, trait neuroticism and the habitual tendency to suppress negative experiences. In combination, these effects hence allow us to argue that measures related to self-defense (e.g. emotional suppression and trait neuroticism) may be intimately linked with social optimism/pessimism. Considering that the misfortune of others can be used to protect and enhance an individual’s self-views (van Dijk *et al*., 2011), the Mode 1 findings could reflect a downsize of future possibilities of distant social out-groups as a boost to perceived self-efficacy. Alternatively, given the potential clinical implications of lower weight measures such as neuroticism, behavioral inhibition, emotion suppression and conscientiousness, one could interpret Mode 1 as a larger and potentially clinically relevant dimension that links the avoidance of potential dangers to self-esteem with basic personality dimensions and social optimism/pessimism measures.

The greatest contributor of the Mode 1 thickness variate was the insula, whose functionality has previously been related to optimistic belief updating (Kuzmanovic *et al*., 2016) and comparative optimism (Blair *et al*., 2013) and thus the preservation of positive personal future-directed expectancies. The present study extends these findings by indicating that the insula is not only functionally but also structurally associated with a dimension across a wide spectrum of optimism bias measures, including primarily social biases. We further observed that the brain regions contributing to Mode 1 largely overlap with those found by fMRI and white matter connectivity studies on optimistic belief updating (Sharot *et al*., 2011; Moutsiana *et al*., 2015). Most important, using the present sample, fMRI analyses in the current experiment revealed striking commonalities between functional and structural aspects of the social optimism bias paradigm (Dricu *et al*., 2020). For instance, when participants assessed event likelihoods for the most distant out-groups (alcoholic character), brain activation differed from assessment of the in-group in the dmPFC, as well as in the IFG pars orbitalis and triangularis (Dricu *et al*., 2020). In sum, the functional literature suggests that the network associated with Mode 1 is primarily linked to self-serving (possibly partly defensive) responding, which is consistent with our interpretation of self-enhancing comparison processes being reflected in the Mode 1 variate (i.e. Mode 1 could be called a ‘defensive self-enhancement dimension with a corresponding insula-inferior frontal cortex substrate’).

The fact that personal biases (comparative optimism and pessimism) were on the same dimension in Mode 1 as social biases for unpopular out-groups (implying an increased likelihood of future misfortune) points toward these biases sharing a biological substrate. However, while this substrate may serve adaptive purposes in promoting biases related to the individual (e.g. necessary condition to keep up task engagement in novel or difficult tasks), it may also interfere with societal efforts aimed at reducing negative stereotyping. Consequently, modifying the magnitude of one type of optimism bias (either personal or social) in a desired direction (e.g. reducing negative stereotypes) may potentially lead to undesired consequences in the other bias (e.g. a reduction of individual optimism and associated task engagement). Because Mode 1 was not only significant but also highly reliable, this mode’s findings can be interpreted as having a strong impact.

We also found significant, but less reliable, canonical correlations between the non-imaging measures and the cortical thickness for Modes 2 and 6, both of which are discussed in more detail in the supplementary online materials. In short, we interpret Mode 2 to represent a particularly dichotomized worldview, bereft of emotional ambiguity. Among other things, this is based on the fact that the overall magnitude of optimism biases was most strongly related to this mode: the larger the biases for the four characters, the greater the cortical thickness. The cortical thickness of Mode 2 pointed to multiple regions throughout the cingulum, which are crucial to both tracking of the stimuli’s subjective value and self-referential processing [e.g. (Cox and Kable, 2014; Yoshimura *et al*., 2014)]. Similar to Mode 1, Mode 2 could be interpreted as being linked to protection of self-value (i.e. Mode 2 could be called a ‘black-and-white valence dimension with a corresponding midline-cingulate substrate’). In Mode 2, however, this would partly be achieved by increasing the influence of affective (perceived warmth) over cognitive (perceived competence) information when evaluating events happening to others with a mixed status [i.e. the businessperson; (Fiske, 2012)].

Mode 6 pointed uniformly to left-sided brain regions and was linked to reduced pessimism and inhibition, but increased optimism biases for members of the in-group and warm out-groups. Like Mode 1 and Mode 2, Mode 6 could be interpreted as relating to the protection of self-value, but with yet another underlying process, that is, by an irrationally strong belief that good things will happen to oneself, the in-group and close out-groups while ignoring possibilities for undesirable events to occur to these individuals (i.e. Mode 6 could hence be called a ‘generalized positivity dimension with a corresponding left hemisphere substrate’). Because this mode seems to neglect biases related to disliked out-groups, it can be considered as (a) reflecting more self-centered (i.e. personal and related to close others) processes than the two other modes and (b) coding positive social aspects of optimism biases.

One limitation of the present study is its correlational approach, implying that we cannot assess causality (i.e. whether variations in social optimism biases and related concepts influence cortical thickness or vice versa). A second limitation is that while Modes 2 and 6 were significant, their reliabilities were limited and need to be replicated. Thus, findings from these two modes should be used for hypothesis generation rather than seen as stable findings.

In conclusion, different facets of social optimism biases (as revealed by our three modes) are associated with increased cortical thickness in a network that includes the left insula, bilateral IFG and dmPFC. Our data suggest that social optimism biases that discriminate against distant out-groups may, to a certain degree, rely on the same biological substrate as personal forms of optimism bias. Thus, a feeling of superiority may be biologically rooted and mediated by different processes: either by up-leveling oneself or by down-leveling disliked others. A mildly irrational optimistic belief in oneself compared with others appears to be healthy, and a lack of it has been associated with psychopathology (Strunk *et al*., 2006; Garrett *et al*., 2014; Korn *et al*., 2014; Blair *et al*., 2017; Dricu *et al*., in press). However, the present study indicates that this belief shares a biological substrate with social biases that can discriminate against out-groups. Brain structure—while plastic—does not change as quickly as brain functionality, underscoring the long-term implications of the present findings. Such implications may well extend beyond neurocognition into fields more directly focused on society at large. Finally, in order to gain better insight into modification possibilities of the different biases, future studies will need to look at causality in the association between diverse facets of optimism and cortical thickness.

*Conflict of interest*: None of the authors had any conflict of interest.

## Funding

This work was supported by the Swiss National Science Foundation, Grants PP00P1_150492 and PP00P1_183709 (to Tatjana Aue), Protocol number: 2015-10-000008. The funders had no role in the study design, data collection and analysis, decision to publish or preparation of the manuscript.

## Supplementary Material

scan-19-324-File006_nsaa095

supplementarydatafile_nsaa095
