# Supplementary material for: Social optimism biases are associated with cortical thickness
Source: Soc Cogn Affect Neurosci. 2020 Jul 18;15(7):745–54. doi: 10.1093/scan/nsaa095 (PMC7511889; doi:10.1093/scan/nsaa095)
Supplement: scan-19-324-File006_nsaa095 [file scan-19-324-file006_nsaa095.docx]

**Supplementary Information**

**Supplementary Methods**

Sparse Canonical Correlation Analysis (sCCA)

Across all 24 non-imaging measures, there was a total of 38 missing data points. All missing data points were replaced with group mean values. All 24 non-imaging and 62 cortical thickness variables (see Supplementary Tables S1A and S1B) were z-standardized across participants and entered into sCCAs implemented in a computer program (MATLAB, version R2017b; MathWorks) by using an in-house script.

For the present analysis, we computed the sparse parameters by running the sCCA with a range of candidate values for the imaging data (from 0.1x√p to 1x√p, at 0.1 increments, where p is the number of features in that view of the data), while no sparsity was implemented on the non-imaging data given its lower number of variables. We selected the optimal sparsity criterion on the basis of which one maximizes the sCCA correlation value. We then computed the optimal sCCA model and determined its significance by using permutations. Accordingly, the imaging data set was permuted 10,000 times before undergoing the exact same analysis as the original data. This permutation procedure was done with the tool Permutation Analysis of Linear Models, freely available at https://fsl.fmrib.ox.ac.uk/fsl/fslwiki/PALM. The p-value was defined as the number of permutations that resulted in a higher correlation than the original data divided by the total number of permutations. Thus, the p-value is explicitly corrected for multiple testing, as it is compared against the null distribution of maximal correlation values across all estimated sCCAs. For each permutation, we tested all sparsity criteria combinations as was done for the original data and then extracted the sCCA correlation with the highest coefficient among the tested options, independent of whether this combination was the same as in the original data. In this way, we ensured that we did not underestimate the chance of a permutation achieving the same or higher value than the original data.

*Reliability analyses*: To ascertain that results from the sCCA were indeed reliable, we conducted three additional analyses:

1. Leave-one-out analyses for each participant. We correlated the identified weights with those of the full data set to verify whether the found solution was strongly skewed by any one participant.

2a. Training-test set analyses. We randomly split the sample in half 10,000 times (resampling), performed sCCA on each of these training sets, and applied the identified weights from each training set to the other half of the sample, that is, the test set. Subsequently, we looked at whether the found correlation would also explain variance in the average test set.

2b. Mean and standard deviation of the redundancy-reliability score (RR-score) for each mode. The RR-score is a measure of the stability of the variable-to-variate correlations and essentially measures whether test sets have similar associations between variables and variates (Moser, 2018; Moser et al., 2018). The code for calculating the RR-score is freely available at <https://www.researchgate.net/publication/329101018_Matlab_code_and_example_to_calculate_RR-score_as_related_to_Moser_et_al_2018>.

3. We tested whether the sample size was adequate or would give rise to gross overfitting. To examine the stability of each sCCA as a function of sample size and composition, we applied a bootstrap resampling approach by randomly resampling 10% to 150% from the original sample (in 10% increments) 1000 times. We then conducted sCCA for the first mode on each bootstrapped subset.

Post hoc analysis: While numerous non-imaging variables correlate with each other (as is expected), this should not lead to multicollinearity problems in sCCA as it would in other methods such as multiple regression, since variance from collinear variables can be combined in its predictiveness and does not necessarily have to be attributed to a single variable.

Supplementary Table S1A: List of non-imaging measures used in sparse canonical correlation analysis.

| Non-imaging measures | Origin | Short description | Significance of a high score |
| --- | --- | --- | --- |
| ERQ Reappraisal | Emotion Regulation Questionnaire (Abler & Kessler, 2009) | Tendency to regulate emotions through the strategy of reappraisal (i.e., reinterpretation of a situation in order to modify its emotional impact) | Strong tendency to use reappraisal as an emotion regulation strategy |
| ERQ Suppression | Emotion Regulation Questionnaire (Abler & Kessler, 2009) | Tendency to regulate emotions through the strategy of suppression (i.e., suppression of behavior and bodily expressions associated with an emotion) | Strong tendency to use suppression as an emotion regulation strategy |
| PANAS Positive | Positive and Negative Affect Schedule (Krohne, Egloff, Kohlmann, & A., 1996; Watson, Clark, & Tellegen, 1988) | Positive affect | Strong positive affect |
| PANAS Negative | Positive and Negative Affect Schedule (Krohne et al., 1996; Watson et al., 1988) | Negative affect | Strong negative affect |
| BFI Extraversion | Big Five Inventory (Rammstedt & John, 2007) | Personality trait “Extraversion” | The participant is extraverted, i.e. likes attention and enjoys meeting other people, among other things |
| BFI Agreeableness | Big Five Inventory (Rammstedt & John, 2007) | Personality trait “Agreeableness” | The participant is high on agreeableness, i.e. tends to be more trusting and altruistic and less competitive, among other things |
| BFI Conscientiousness | Big Five Inventory (Rammstedt & John, 2007) | Personality trait “Conscientiousness” | The participant is conscientious, i.e. tends to be prepared and enjoy having a set schedule, among other things |
| BFI Neuroticism | Big Five Inventory (Rammstedt & John, 2007) | Personality trait “Neuroticism” | The participant is neurotic, i.e. tends to easily experience a lot of stress, anxiety, and worry, among other things |
| BFI Openness | Big Five Inventory (Rammstedt & John, 2007) | Personality trait “Openness” | The participant is open, i.e. tends to be creative and like new ideas and abstract concepts, among other things |
| RSES Total | Rosenberg Self-Esteem Scale (Rosenberg, 1965; Von Collani & Herzberg.Y, 2003) | Self-esteem, a concept associated with optimism (see correlations with LOT and COS in supplemental data) | The participant is self-assured, i.e. likely to believe that she or he will succeed if undertaking a task |
| BIS Total | Behavioral Inhibition and Behavioral Activation System Scales (Carver & White, 1994; Strobel, Beauducel, Debener, & Brocke, 2001) | Regulation of aversive motives; sensitivity to punishment; tendency for nervousness and inhibition of behavioral interactions | Strong inhibition, i.e. tendency to be nervous and inhibited, among other things |
| BAS Total | Behavioral Inhibition and Behavioral Activation System Scales (Carver & White, 1994; Strobel et al., 2001) | Regulation of appetitive motives; sensitivity to reward; tendency to approach goals; outgoing behavior | Strong sensitivity for and approach to reward, i.e. tendency to approach goals directly and be outgoing, among other things |
| COS Optimism | Comparative Optimism Scale (Weinstein, 1980) | Personal belief of likelihood for desirable events/successes to happen to the participant compared with others of same age and sex | The participant thinks good things are more likely to happen to him/her than others |
| COS Pessimism | Comparative Optimism Scale (Weinstein, 1980) | Personal belief of likelihood for undesirable events/failures to happen to the participant compared with others of same age and sex | The participant thinks bad things are more likely to happen to him/her than others |
| LOT Optimism | Revised Life Orientation Test (Glaesmer, Hoyer, Klotsche, & Herzberg, 2008; Scheier, Carver, & Bridges, 1994) | Dispositional optimism | The participant thinks good things are likely to happen to him/her |
| LOT Pessimism | Revised Life Orientation Test (Glaesmer et al., 2008; Scheier et al., 1994) | Dispositional pessimism | The participant thinks bad things are likely to happen to him/her |
| Student optimism bias | Social Optimism Bias Task | Difference between the average likelihood that a participant assigned to desirable vs. undesirable events for the student character | The participant thinks desirable events are more likely to happen to the student than undesirable events |
| Elderly optimism bias | Social Optimism Bias Task | Difference between the average likelihood that a participant assigned to desirable vs. undesirable events for the elderly person character | The participant thinks desirable events are more likely to happen to the elderly person than undesirable events |
| Businessperson optimism bias | Social Optimism Bias Task | Difference between the average likelihood that a participant assigned to desirable vs. undesirable events for the businessperson character | The participant thinks desirable events are more likely to happen to the businessperson than undesirable events |
| Alcoholic optimism bias | Social Optimism Bias Task | Difference between the average likelihood that a participant assigned to desirable vs. undesirable events for the alcoholic character | The participant thinks desirable events are more likely to happen to the alcoholic person than undesirable events |
| Magnitude of optimism bias | Social Optimism Bias Task | Average of optimism bias for all characters, with the value of the alcoholic character being inversed (due to being negative on average) | The participant tends toward a strong optimism bias across characters (low values: participant tends toward small bias values across characters) |
| Desirable events likelihood average | Social Optimism Bias Task | Average likelihood of desirable events that a participant gives across all characters | The participant thinks that desirable events are very likely to happen to all characters |
| Undesirable events likelihood average | Social Optimism Bias Task | Average likelihood of undesirable events that a participant gives across all characters | The participant thinks that undesirable events are very likely to happen to all characters |
| Warmth bias | Social Optimism Bias Task | Sum of optimism biases of the student and elderly characters minus sum of the optimism biases of the businessperson and alcoholic characters | The participant thinks that desirable events are more likely to happen to students and elderly persons than to businesspersons and alcoholic persons, while undesirable events are less likely to happen to students and elderly persons than to businesspersons and alcoholic persons. |

*Note.* Abbreviations: BAS = Behavioral Activation System Scale, BIS = Behavioral Inhibition System Scale, BFI = Big Five Inventory, COS = Comparative Optimism Scale, ERQ = Emotion Regulation Questionnaire, LOT = Life Orientation Test, PANAS = Positive and Negative Affect Schedule, RSES = Rosenberg Self-Esteem Scale.

Supplementary Table 1B: List of cortical thickness regions entered as measures into sparse canonical correlation analysis.

| Cortical thickness measures (each existing once for the right and once for the left hemisphere) | |
| --- | --- |
| Medial orbitofrontal cortex | Transverse temporal gyrus |
| Lateral orbitofrontal cortex | Supramarginal gyrus |
| Inferior frontal gyrus (pars opercularis) | Entorhinal cortex |
| Inferior frontal gyrus (pars orbitalis) | Parahippocampal gyrus |
| Inferior frontal gyrus (pars triangularis) | Inferior temporal gyrus |
| Caudal middle frontal gyrus | Middle temporal gyrus |
| Rostral middle frontal gyrus | Superior temporal gyrus |
| Superior frontal gyrus | Fusiform gyrus |
| Precentral gyrus | Lingual gyrus |
| Paracentral lobule | Precuneus |
| Postcentral gyrus | Cuneus |
| Rostral anterior cingulate cortex | Inferior parietal lobe |
| Caudal anterior cingulate | Superior parietal lobe |
| Posterior cingulate cortex (above corpus callosum) | Pericalcarine sulcus |
| Isthmus of the cingulate | Lateral occipital gyrus |
| Insula |  |


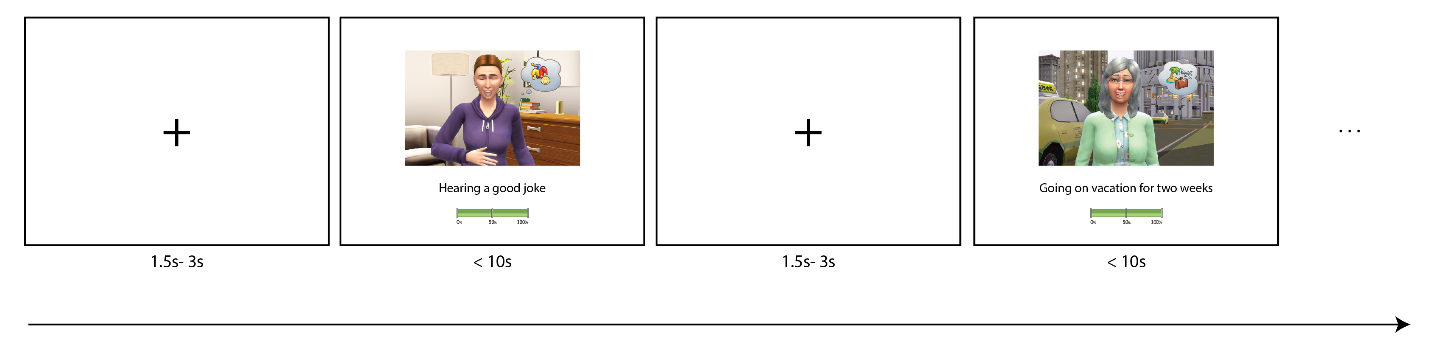


Supplementary Figure S1: Layout of the experimental design. Following the on-screen introduction of the four fictional characters and the explanation of the task, participants rated the likelihood estimates of each character who experienced each of the 32 target events. Each of the 128 trials began with a fixation cross with a jittered duration of 1.5 s to 3 s, followed by a single screen depicting the target character in the target event, a one-sentence description of the target event, and the visual analog scale ranging from 0% to 100%.

Supplementary Table S2: List of events used.

|  | Event | Desirability | Frequency | Controllability |
| --- | --- | --- | --- | --- |
| 1 | Being hugged ^a^ | High | High | High |
|  | A friend returns borrowed money to you ^b^ | High | High | High |
|  | Being greeted warmly by relatives at a family celebration ^b^ | High | High | High |
|  | Enjoy a warm bath or shower on a cold day ^b^ | High | High | High |
| 2 | Hearing a very funny joke ^b^ | High | High | Low |
|  | New neighbor comes over to introduce themselves ^b^ | High | High | Low |
|  | A song that you like comes on the radio ^b^ | High | High | Low |
|  | Bumping into an old friend on the street ^b^ | High | High | Low |
| 3 | Delivering a speech successfully ^a^ | High | Low | High |
|  | A child has fun while you take care of him for 2 hours ^a^ | High | Low | High |
|  | Winning a karaoke contest ^a^ | High | Low | High |
|  | Writing a bestseller about one’s own life ^a^ | High | Low | High |
| 4 | Win a car in the lottery ^b^ | High | Low | Low |
|  | Find a 20 CHF bill on the ground ^b^ | High | Low | Low |
|  | Seeing a comet in the sky ^b^ | High | Low | Low |
|  | Win a sports bet ^b^ | High | Low | Low |
| 5 | The store closes just as you arrive ^b^ | Low | High | High |
|  | Finding rotten food in the refrigerator ^b^ | Low | High | High |
|  | Using a public restroom ^b^ | Low | High | High |
|  | Drinking cold coffee/tea ^a^ | Low | High | High |
| 6 | A neighbor is listening to very loud music ^b^ | Low | High | Low |
|  | Getting heartbroken after a relationship ^b^ | Low | High | Low |
|  | Being confused with another person ^b^ | Low | High | Low |
|  | Computer crashes in the middle of writing a text ^b^ | Low | High | Low |
| 7 | Marriage ends in a bitter divorce ^b^ | Low | Low | High |
|  | Lose 50 CHF ^a^ | Low | Low | High |
|  | Developing an excruciating toothache ^b^ | Low | Low | High |
|  | Suffering a wound that needs to be sewn ^b^ | Low | Low | High |
| 8 | Witnessing a robbery of a bank ^b^ | Low | Low | Low |
|  | Being falsely accused of a serious crime ^b^ | Low | Low | Low |
|  | Receiving a dog bite ^b^ | Low | Low | Low |
|  | Witnessing a tree falling on a house ^b^ | Low | Low | Low |

*Note*. Four examples for each possible combination of valence, frequency, and controllability were used as indicated in previous literature (Dricu et al., 2018).

^a^ Scenarios from (Dricu et al., 2018). ^b^ Scenarios adapted from (Chambers, Windschitl, & Suls, 2003).

**Supplementary Results**

An excel file with supplementary data can be found on github at: <https://github.com/domamo/Supplementaries-Social-optimism-biases-are-associated-with-cortical-thickness>


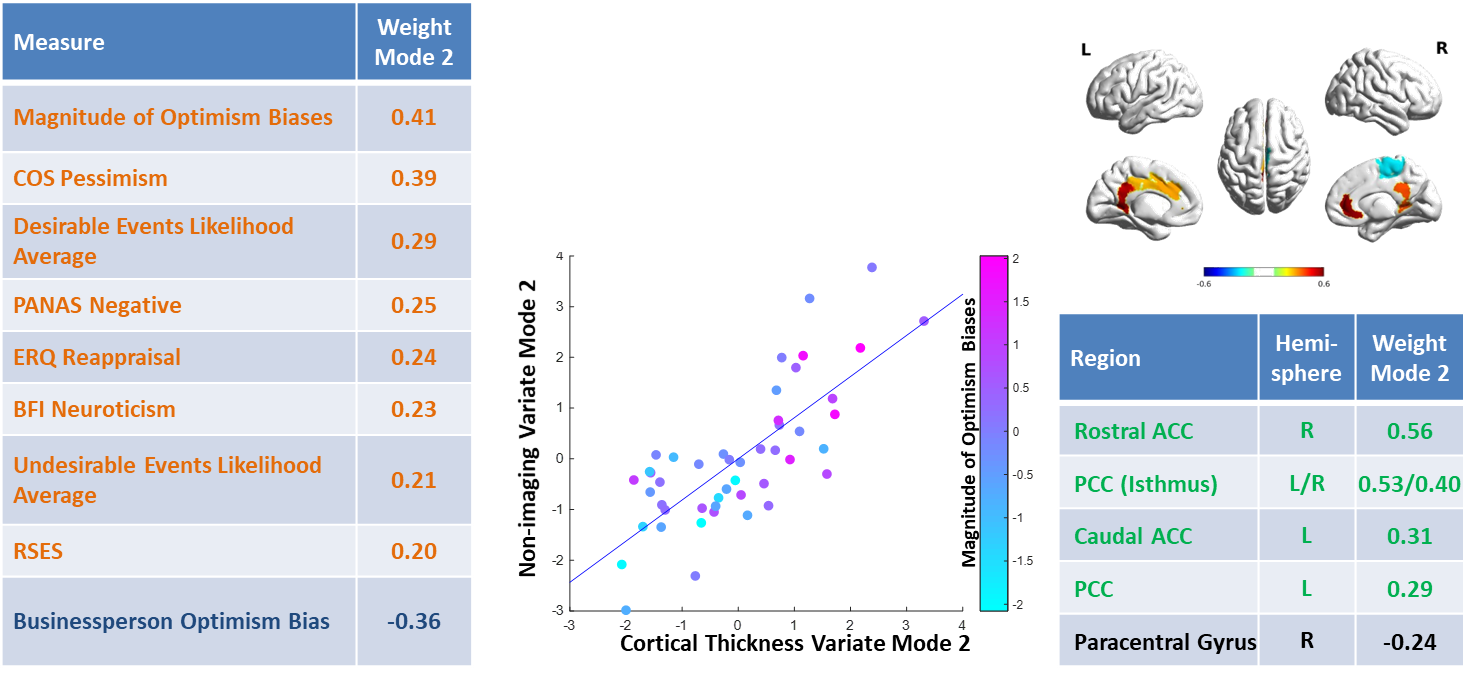


Supplementary Figure S2: Depiction of results of the sparse canonical correlation analysis Mode 2. Left: List of all non-imaging variables with weights above 0.2 in descending order. Middle: Scatterplot of both variates (x-axis: cortical thickness, y-axis: non-imaging data) colored by the z-standardized magnitude of optimism bias across different characters. Top right: Depiction of the weights of the brain regions with contributions toward the imaging variate above 0.2. Bottom right: List of these weights in descending order. Abbreviations: ACC = anterior cingulate cortex, BFI = Big Five Inventory, COS = Comparative Optimism Scale, ERQ = Emotion Regulation Questionnaire, PANAS = Positive and Negative Affect Schedule, PCC = posterior cingulate cortex, RSES = Rosenberg Self-Esteem Scale.

**
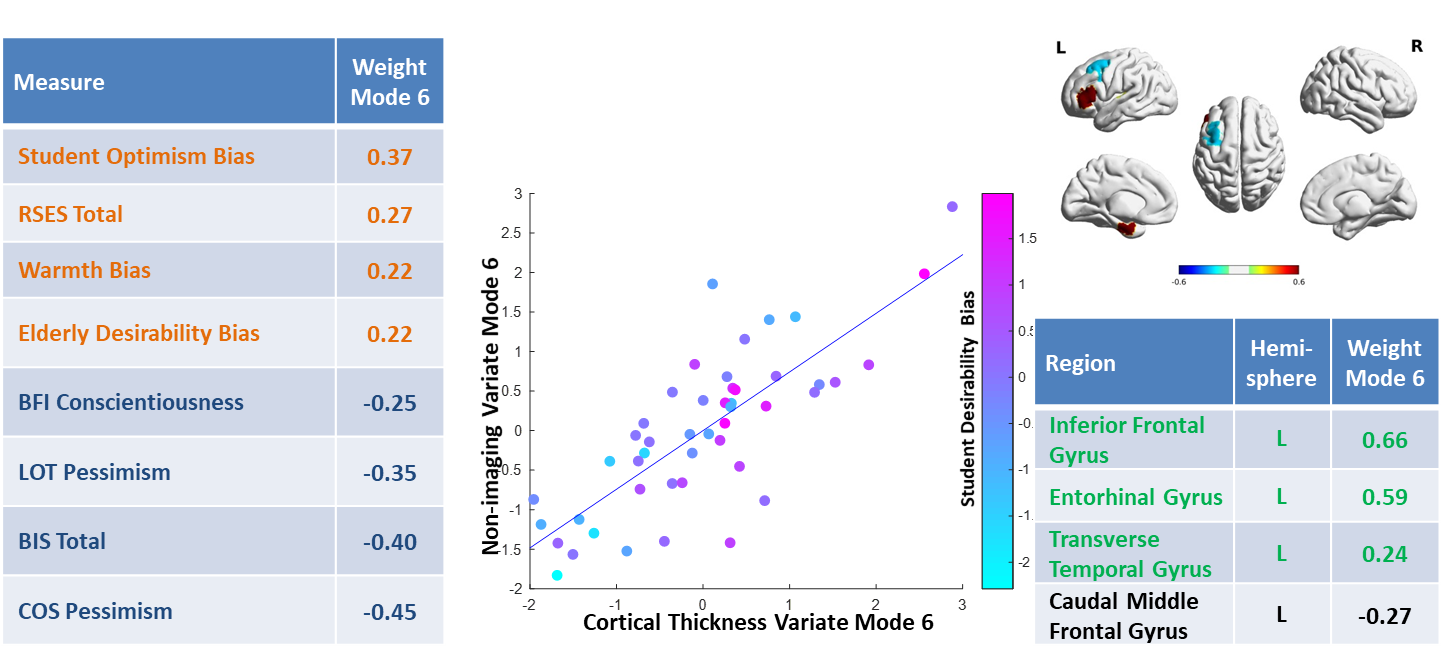
**

Supplementary Figure S3: Depiction of results of the sparse canonical correlation analysis Mode 6. Left: List of all non-imaging variables with weights above 0.2 in descending order. Middle: Scatterplot of both variates (x-axis: cortical thickness, y-axis: non-imaging data) colored by the z-standardized student optimism bias. Top right: Depiction of the weights of the brain regions with contributions toward the imaging variate above 0.2. Bottom right: List of these weights in descending order. Abbreviations: BFI = Big Five Inventory, BIS = Behavioral Inhibition System Scale, COS = Comparative Optimism Scale, LOT = Life Orientation Test, RSES = Rosenberg Self-Esteem Scale.

**Post-hoc analysis**


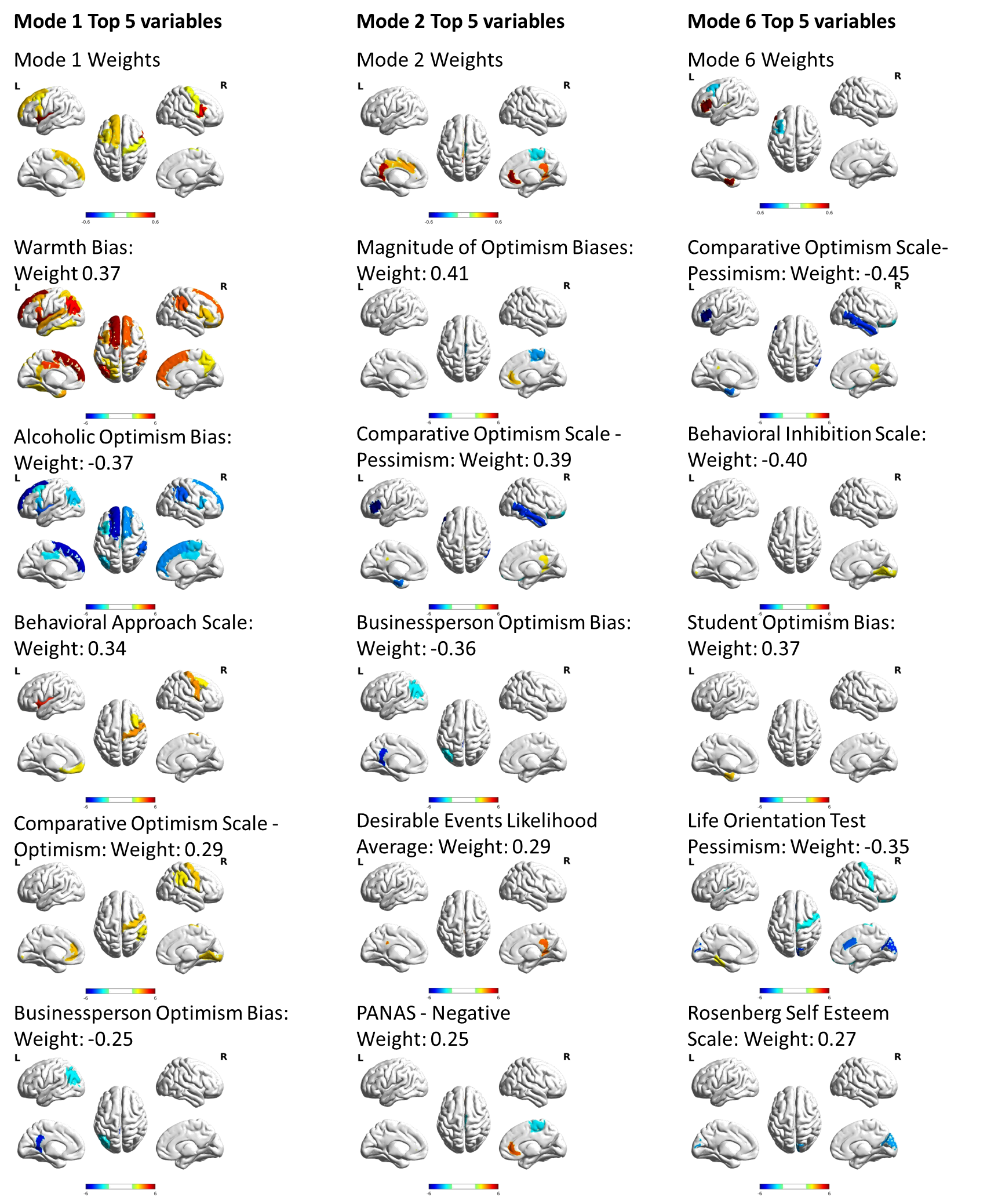


Supplementary Figure S4: Top five variables of each significant mode correlated with cortical thickness. The scale is the inversed logarithm of the p-values. The regions depicted are p uncorrected < 0.001. Abbreviations: PANAS = Positive and Negative Affect Schedule.

**Reliability Analysis**

Leave-one-out analyses indicated that no single participant was an outlier, as weights for each analysis correlated at r > 0.84 in each case. Training-test set analysis indicated that Mode 1 had a median correlation of r = 0.26 in test sets, while Modes 2 (median r = 0.01) and 6 (median r = 0.05) had very low correlations in test sets. RR-scores also indicated good reliability for Mode 1 (mean RR-score = 0.69, SD = 0.10), while those of Modes 2 (mean RR-score = 0.37, SD = 0.17) and 6 (mean RR-score = 0.23, SD = 0.12) indicated considerably lower reliability (see Supplementary Figure S5B and supplementary data set). Sample size testing indicated that there was only a slight amount of overfitting and general stabilization of the effect at about 80% of the sample size, and doubling the sample size would have diminished the average sCCA correlation from 0.78 to 0.76 (see Supplementary Figure S5C).


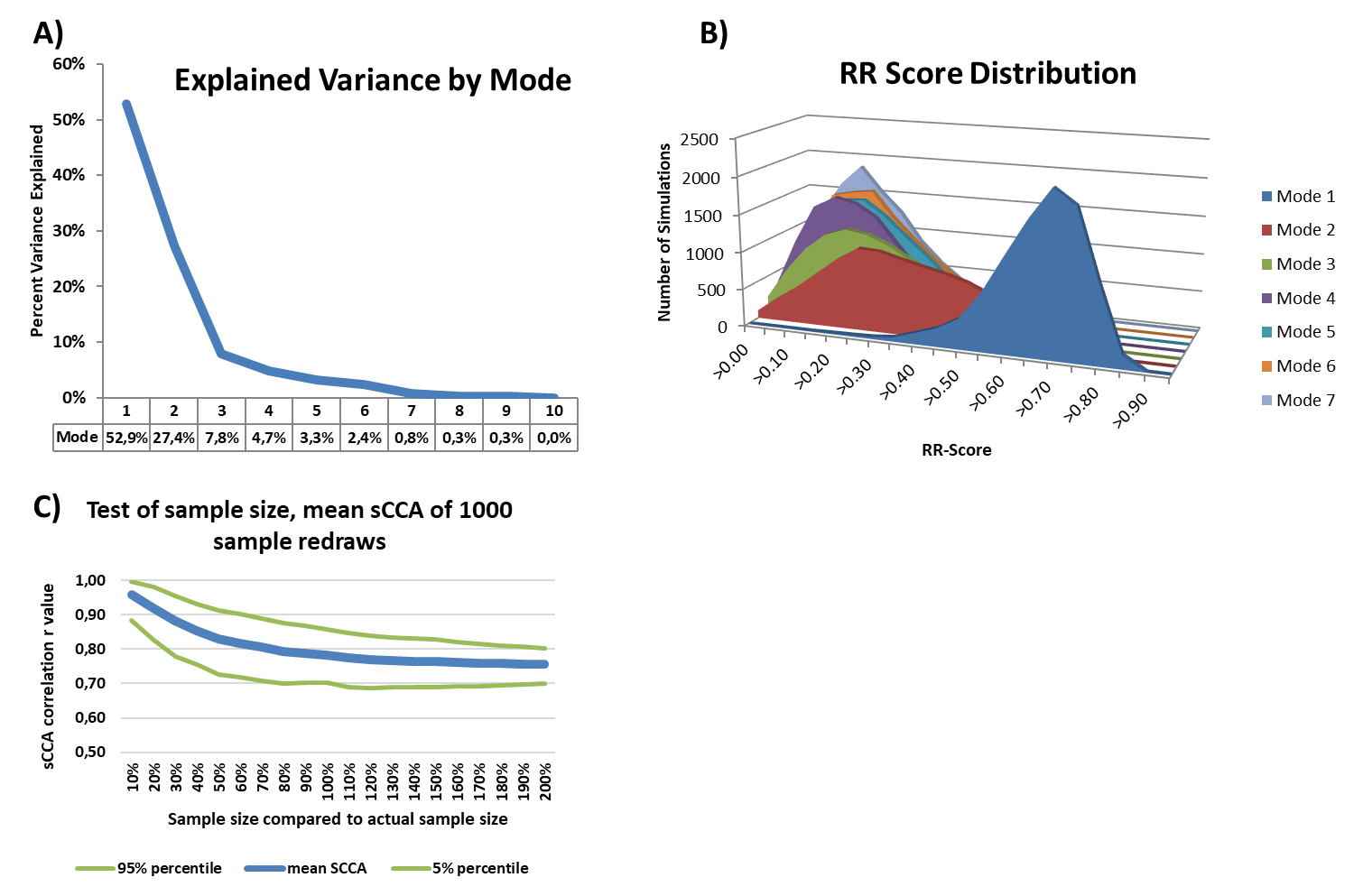


Supplementary Figure S5: A) Depiction of explained variance by mode (component pair) of the sparse canonical correlation analysis. B) Distribution of redundancy-reliability scores (RR-scores) across modes. C) Estimation of overfitting by the sparse canonical correlation analysis, done via randomly redrawing subsamples (or oversamples) and reperforming the analysis. The figure displays the average correlation shown.

**Supplementary Discussion**

Mode 2 is most strongly related to an increased magnitude of the overall optimism bias displayed by a participant (across all four characters). It further involves the attribution of high likelihoods to both undesirable and desirable events, relating to a participant’s overall response tendency (i.e., tendency to give either high or low likelihood estimates). Notably, even if an individual believed that all kinds of events frequently occurred, there can still be systematic differences between undesirable and desirable events, thereby allowing for the appearance of strong optimism biases (reflected in the magnitude bias being the main contributor to Mode 2). Therefore, such extremist thinking may permit a particularly dichotomized worldview. One explanation for such thinking may relate to increased trait neuroticism (BFI Neuroticism scale; increased worrying and experience of stress). Together with the fact that the Mode 2 variate involves a positive weight for both comparative (i.e., self-centered) pessimism (COS Pessimism scale) and negative affect (PANAS Negative scale), this points to the possibility that the mode’s bias dimension is defensive in nature.

In the context of such an interpretation, the negative weight revealed for the businessperson optimism bias (i.e., increased Mode 2 variate scores for less optimistic expectancies for the businessperson) may be of interest. Previous literature on competent-but-cold individuals, which includes businesspeople, indicates a more ambivalent treatment, with only slightly optimistic optimism biases (Fiske, 2012): Perceived competence should translate into the capacity to maximize desirable outcomes and minimize undesirable outcomes, but these optimal effects are muddled and greatly reduced by the perceived cold traits (which are associated with the reverse effects). It appears that the more the cold rather than the competence traits influence the optimism bias (toward decreasing optimism) in participants, the higher the cortical thickness of the biological correlate that was simultaneously associated with overall magnitude of bias and a more extremist worldview (as indicated by generally high likelihoods). Interestingly, the social biases related to the remaining characters (student, elderly, alcoholic) were not related to the Mode 2 variate. Therefore, brain structure in the cingulum (ACC and PCC) appears to be specifically linked to the assessment of ambiguous out-groups toward which there is moderate social distance. This moderate social distance may go along with an increased uncertainty about how to treat that group and how to relate that information to the self (Fiske, 2012).

In addition, Mode 2 can be interpreted in terms of self-referential processing, as indicated, for example, by the contribution of comparative pessimism and self-esteem (RSES), both of which depend on an individual’s belief that she or he will succeed at tasks. The association of Mode 2 with self-referential processing could further be inferred through the brain regions that contribute – that is, almost the entire cingulate cortex, different parts of which have been linked to self-referential processing (Herold, Spengler, Sajonz, Usnich, & Bermpohl, 2016) and subjective value tracking (Cox & Kable, 2014) – to optimism as a trait (Sharot, Riccardi, Raio, & Phelps, 2007) and also to the evaluation of in-group-members and to stimulus evaluation in general (Cikara, Van Bavel, Ingbretsen, & Lau, 2017; Maddock, Garrett, & Buonocore, 2001; Morrison, Decety, & Molenberghs, 2012; Volz, Kessler, & von Cramon, 2009). In sum, similar to Mode 1, Mode 2 can be interpreted to reflect the protection of self-value, although through a different mechanism. In Mode 2, this might happen by eliminating ambiguity in stimulus evaluation. Overall, Mode 2 could be considered a “black-and-white valence dimension with a corresponding midline-cingulate substrate.”

Mode 6 is the one that is most closely related to biases centered around the self and close others. The positive sign of the weight for warmth bias likely results from both the student and the elderly character biases (i.e., the in-group and the close “warm” out-group) contributing positively to this mode. Thus, this mode codes positive social aspects of optimism biases (rather than diminishing tendencies toward the more negatively evaluated out-group businessperson and alcoholic characters). Consistent with this interpretation, self-esteem (RSES) was positively implicated in this mode. Behavioral inhibition (BIS scale) contributed negatively, signaling a decrease of withdrawal or avoidance tendencies with stronger expressions of the Mode 6 variate. Moreover, both the weights for trait pessimism (LOT Pessimism scale) and comparative pessimism (COS Pessimism scale) were negative, suggesting the mode’s implication relies in part on a decrease in self-centered pessimistic tendencies.

All brain regions contributing to Mode 6 were located in the left hemisphere. The left hemisphere has previously been linked to stronger optimistic outlooks on life than has the right hemisphere (Hecht, 2013), including related concepts such as higher self-esteem (De Raedt, Franck, Fannes, & Verstraeten, 2008) and increased feelings of the self being the locus of control, particularly in reference to semantic matching tasks (De Brabander, Boone, & Gerits, 1992). This link with language processing skills and self-esteem is supported by the fact that the regions that contribute most to our Mode 6 are the IFC (pars triangularis; part of Broca’s area) and the left entorhinal cortex (important in declarative memory (Naya, 2016)). Like Mode 1 and Mode 2, Mode 6 could be interpreted to be related to the protection of the self, but with yet another underlying process, that is, by an irrationally strong belief that good things will happen to oneself, the in-group and close out-groups, while ignoring the risks for undesirable events to occur. One might hypothesize that such a mode would allow one to take on tasks that may seem (socially) risky. Overall, Mode 6 could be considered a “generalized positivity dimension with a corresponding left hemisphere substrate.”

**Supplementary References**

Abler, B., & Kessler, H. (2009). Emotion Regulation Questionnaire -Eine deutschsprachige Fassung des ERQ von Gross und John. *Diagnostica, 55*, 142-152.

Carver, C. S., & White, T. L. (1994). Behavioral-Inhibition, Behavioral Activation, and Affective Responses to Impending Reward and Punishment - the Bis Bas Scales. *Journal of Personality and Social Psychology, 67*(2), 319-333. doi:Doi 10.1037//0022-3514.67.2.319

Chambers, J. R., Windschitl, P. D., & Suls, J. (2003). Egocentrism, event frequency, and comparative optimism: When what happens frequently is “more likely to happen to me”. *Personality and Social Psychology Bulletin, 29*(11), 1343-1356.

Cikara, M., Van Bavel, J. J., Ingbretsen, Z. A., & Lau, T. (2017). Decoding “us” and “them”: Neural representations of generalized group concepts. *Journal of Experimental Psychology: General, 146*(5), 621.

Cox, K. M., & Kable, J. W. (2014). BOLD subjective value signals exhibit robust range adaptation. *J Neurosci, 34*(49), 16533-16543. doi:10.1523/JNEUROSCI.3927-14.2014

De Brabander, B., Boone, C., & Gerits, P. (1992). Locus of control and cerebral asymmetry. *Percept Mot Skills, 75*(1), 131-143. doi:10.2466/pms.1992.75.1.131

De Raedt, R., Franck, E., Fannes, K., & Verstraeten, E. (2008). Is the relationship between frontal EEG alpha asymmetry and depression mediated by implicit or explicit self-esteem? *Biol Psychol, 77*(1), 89-92. doi:10.1016/j.biopsycho.2007.06.004

Dricu, M., Buhrer, S., Hesse, F., Eder, C., Posada, A., & Aue, T. (2018). Warmth and competence predict overoptimistic beliefs for out-group but not in-group members. *PLoS One, 13*(11), e0207670. doi:10.1371/journal.pone.0207670

Fiske, S. T. (2012). Managing ambivalent prejudices: smart-but-cold and warm-but-dumb stereotypes. *The Annals of the American Academy of Political and Social Science, 639*(1), 33-48.

Glaesmer, H., Hoyer, J., Klotsche, J., & Herzberg, P. Y. (2008). Die deutsche Version des Life-Orientation-Test (LOT-R) zum dispositionellen Optimismus und Pessimismus. *Zeitschrift für Gesundheitspsychologie, 16*, 26-31.

Hecht, D. (2013). The neural basis of optimism and pessimism. *Exp Neurobiol, 22*(3), 173-199. doi:10.5607/en.2013.22.3.173

Herold, D., Spengler, S., Sajonz, B., Usnich, T., & Bermpohl, F. (2016). Common and distinct networks for self-referential and social stimulus processing in the human brain. *Brain Struct Funct, 221*(7), 3475-3485. doi:10.1007/s00429-015-1113-9

Krohne, H. W., Egloff, B., Kohlmann, C. W., & A., T. (1996). Untersuchungen mit der deutechen Version "Positive and Negativee Affect Schedule" (PANAS). *Diagnostica, 42*, 139-156.

Maddock, R. J., Garrett, A. S., & Buonocore, M. H. (2001). Remembering familiar people: the posterior cingulate cortex and autobiographical memory retrieval. *Neuroscience, 104*(3), 667-676.

Morrison, S., Decety, J., & Molenberghs, P. (2012). The neuroscience of group membership. *Neuropsychologia, 50*(8), 2114-2120.

Moser, D. A. (2018). Matlab code and example to calculate RR-score as related to Moser et al 2018 (Publication no. 10.13140/RG.2.2.33544.26883).

Moser, D. A., Doucet, G. E., Lee, W. H., Rasgon, A., Krinsky, H., Leibu, E., . . . Frangou, S. (2018). Multivariate Associations Among Behavioral, Clinical, and Multimodal Imaging Phenotypes in Patients With Psychosis. *JAMA Psychiatry, 75*(4), 386-395. doi:10.1001/jamapsychiatry.2017.4741

Naya, Y. (2016). Declarative association in the perirhinal cortex. *Neurosci Res, 113*, 12-18. doi:10.1016/j.neures.2016.07.001

Rammstedt, B., & John, O. P. (2007). Measuring personality in one minute or less: A 10-item short version of the Big Five Inventory in English and German. *Journal of Research in Personality, 41*(2007), 203–212. doi:doi:10.1016/j.jrp.2006.02.001

Rosenberg, M. (1965). *Society and the adolescent selfimage*. Princeton, NJ: Princeton University Press.

Scheier, M. F., Carver, C. S., & Bridges, M. W. (1994). Distinguishing Optimism from Neuroticism (and Trait Anxiety, Self-Mastery, and Self-Esteem) - a Reevaluation of the Life Orientation Test. *Journal of Personality and Social Psychology, 67*(6), 1063-1078. doi:Doi 10.1037/0022-3514.67.6.1063

Sharot, T., Riccardi, A. M., Raio, C. M., & Phelps, E. A. (2007). Neural mechanisms mediating optimism bias. *Nature, 450*(7166), 102.

Strobel, A., Beauducel, A., Debener, S., & Brocke, B. (2001). A German Version of Carver and White's BIS/BAS Scales. *Zeitschrift für Differentielle und Diagnostische Psychologie, 22*(3), 216-227.

Volz, K. G., Kessler, T., & von Cramon, D. Y. (2009). In-group as part of the self: in-group favoritism is mediated by medial prefrontal cortex activation. *Social neuroscience, 4*(3), 244-260.

Von Collani, G., & Herzberg.Y. (2003). Eine revidierte Fassung der deutschsprachigen Skala zum Selbstwertgefühl von Rosenberg. *Zeitschrift für Differentielle und Diagnostische Psychologie, 24*(1), 3-7.

Watson, D., Clark, L. A., & Tellegen, A. (1988). Development and validation of brief measures of positive and negative affect: the PANAS scales. *J Pers Soc Psychol, 54*(6), 1063-1070.

Weinstein, N. D. (1980). Unrealistic optimism about future life events. *Journal of Personality and Social Psychology, 39*(806-820). doi:10.1037/0022-3514.39.5.806
